# Optically controllable magnetism in atomically thin semiconductors

**DOI:** 10.1126/sciadv.abq7650

**Published:** 2022-09-30

**Authors:** Kai Hao, Robert Shreiner, Andrew Kindseth, Alexander A. High

**Affiliations:** ^1^ Pritzker School of Molecular Engineering, University of Chicago , Chicago, IL 60637, USA.; ^2^ Department of Physics, University of Chicago, Chicago, IL 60637, USA.; ^3^ Center for Molecular Engineering and Materials Science Division, Argonne National Laboratory, Lemont, IL 60439, USA.

## Abstract

We report evidence that ferromagnetic order in electrostatically doped, monolayer transition metal dichalcogenide (TMD) semiconductors can be stabilized and controlled at zero magnetic field by local optical pumping. We use circular dichroism (CD) in reflectivity from excitonic states as a spatially resolved probe of charge-carrier spin polarization. At electron densities *n_e_* ~ 10^12^ cm^−2^, a diffraction-limited, circularly polarized optical pump breaks symmetry between oppositely polarized magnetic states and stabilizes long-range magnetic order, with carrier polarization exceeding 80% over an 8 μm by 5 μm extent. In time-resolved measurements with pulsed optical excitation, we observe that magnetic interactions amplify the initial pump-induced spin polarization by more than an order of magnitude. The optical control of magnetism with local optical pumps will unlock advancements in spin and optical technologies and provides a versatile tool in the study of correlated phases in two-dimensional electron gases.

## Teaser

Local optical pumping stabilizes long-range magnetic order in a two-dimensional semiconductor.

## INTRODUCTION

Interacting electrons in two-dimensional (2D) electron gases (2DEGs) can exhibit a remarkable variety of correlated phases including Wigner crystals, Mott insulators, charge density waves, and magnetism (*1*–*6*). Because of favorable material properties and tuning capabilities, transition metal dichalcogenides (TMDs) are a rapidly emerging platform for the study and manipulation of 2DEGs. The interaction strength is typically characterized by the dimensionless parameter *r*_s_ ~ $m/(\varepsilon \sqrt{n})\;$, where ε is the permittivity, *n* is the electron density, and *m* is the effective mass. For free electrons in TMDs, the combination of large effective mass ~0.44 *m*_e_ and reduced dielectric screening creates a Bohr radius that is only slightly larger than the lattice constant, yielding *r*_s_ values exceeding 20 at experimentally accessible densities of 10^11^ to 10^12^ cm^−2^ (*5*, *7*–*9*). As a result, the energy of Coulombic interactions can be appreciably larger than energies associated with phase-space filling, leading to collective ordering of electronic states dictated by long-range exchange interactions (*3*–*6*, *10*–*18*). In particular, exchange interactions are predicted to create a variety of spin- and valley-polarized itinerant magnetic phases (*11*–*13*).

Recently, experiments have shown that under applied magnetic fields and in certain doping regimes, electrons in molybdenum disulfide (MoS_2_) and molybdenum diselenide (MoSe_2_) exhibit magnetic order with near-complete spin polarization far beyond the predictions of a simple thermal population model (*7*, *8*, *18*). The spin polarization manifests as circular dichroism (CD) in reflectivity and photoluminescence (PL) measurements of the excitonic states and was initially attributed to either interaction-enhanced electronic *g*-factor (so-called giant paramagnetism) (*18*) or the emergence of ferromagnetic order (*7*, *8*). In the ferromagnetism model, the spin polarization is due to strong exchange interactions, which favor the formation of a spin-polarized state in both the K and K′ valleys (*7*, *13*). Follow-up experiments demonstrated that the system transitions from a ferromagnetic to a paramagnetic phase with increased doping, suggesting direct electronic control over the electron-electron interactions and correlated phases (*8*). These studies present compelling evidence that the magnetic ordering is ferromagnetic in nature. However, no net magnetization or spin polarization was observed at zero applied magnetic field. This absence was attributed to fluctuating nanoscale domains and the lack of a global symmetry breaking mechanism (*8*).

Optical pumping is a conceivable mechanism for breaking the symmetry between equivalent spin configurations in TMDs. Recent studies have shown that pumping individual monolayers or heterostructures of TMDs with circularly polarized light can generate spin imbalances with microsecond-long relaxation times (*19*, *20*). For WSe_2_ monolayers in the electron-doped region, which is the main focus of this work, resident electrons can be dynamically spin-/valley-polarized by continuous pumping with circular light (*21*). Photo-generated electrons excited in a selected valley by the circularly polarized pump will preferentially relax to the opposite valley due to fast spin-conserving intervalley scattering. In addition, the intravalley recombination of conduction electrons with photo-generated holes forming dark excitons can enhance the asymmetry of the valley populations (*20*). The resulting spin polarization is maintained in the presence of the continuous pump as these processes occur on time scales faster than the spin relaxation rate (*20*, *21*). Moreover, due to the relatively low free-charge carrier densities *n* ~ 10^12^ cm^−2^, a substantial population of resident carriers may be spin-polarized, potentially sufficient to break the symmetry between ground-state spin configurations and stabilize magnetic order in alignment with the pumped spins.

## RESULTS

**Fig. 1.**
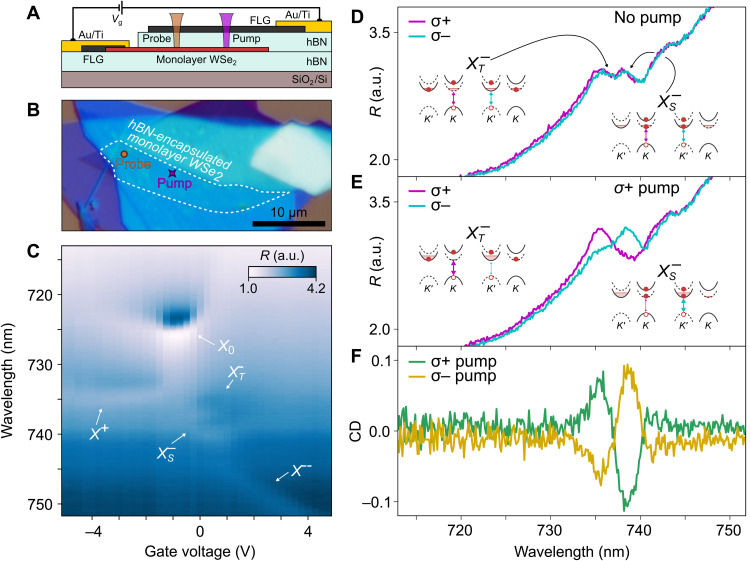
Sample under study. (**A**) Schematic of hBN-encapsulated WSe_2_ monolayer with FLG top gate and contacts. The optical pump and probe are spatially separated. (**B**) Optical microscope image of sample D1. (**C**) Gate-dependent reflection spectra of the WSe_2_ sample. The excitonic resonance features are labeled correspondingly. a.u., arbitrary units. (**D**) σ^+^ and σ^−^ reflection spectra at 0.5 V, where the singlet and triplet trion features are well resolved. Inset: Singlet and triplet trion configurations showing balanced valley populations. Solid and dashed bands indicate spin ordering. (**E**) σ^+^ and σ^−^ reflection spectra at 0.5 V (*n_e_* ~2 × 10^12^cm^−2^) under σ^+^ pumping. Insert: Schematic of singlet and triplet trions in optically pumped spin/valley-polarized electron bath. (**F**) CD spectra under σ^+^ and σ^−^ pumping. Note that *T* = 4 K, pump power is 7.8 μW, and pump-probe offset is 8 μm.

Here, we study the impact of above-bandgap, circularly polarized optical pumping on hexagonal boron nitride (hBN) encapsulated monolayers of WSe_2_, showing evidence of optically stabilized, nonlocal magnetic order. The heterostructure layout and optical image of the sample D1 are presented in Fig. 1 (A and B). The doping level in the monolayer can be controlled by applying gate voltage between the few-layer graphene (FLG) contact and the top gate and manifests in the appearance of neutral and charged excitonic resonances in the reflection spectra (Fig. 1C). We first focus on low-temperature measurements at *T* = 4 K within the moderately electron-doped region, where bound singlet (X^−^_S_) and triplet (X^−^_T_) trions are observed (*22*–*28*). Reflection from a circularly polarized supercontinuum laser provides a probe of the local, valley-selective optical response. In the absence of pumping, the balanced reflection of σ^+^- and σ^−^-polarized light is observed (Fig. 1D). Next, we pump the sample with a 660-nm diffraction-limited continuous wave (CW) laser with a submicrometer spot size and a power of 7.8 μW. To demonstrate the nonlocality of pump-induced effects and to eliminate the influence of PL in detection, the probe spot is separated by nearly 8 μm from the pump (Fig. 1B). The reflection spectra under σ^+^-polarized pumping are markedly different—the triplet (singlet) trion dominates the probe signal co(cross)–polarized to the pump (Fig. 1E). This pump-induced CD is characterized by CD *=* Δ*R^+^ −* Δ*R^−^*, where Δ*R^+,−^* = (*R^+,−^*_on_/*R*^*+,*−^_off_) – 1 is the differential reflectivity comparing the σ*^+,−^* probed reflection in the presence (*R^+,−^*_on_) and absence (*R^+,−^*_off_) of the pump. The CD signal displays amplitudes approaching 10% and inverts with the sign of the pump polarization (Fig. 1F).

CD is a direct signature of electron spin/valley polarization and has been used to study spin imbalances in a range of materials and doping regimes (*7*, *18*, *19*, *29*–*31*). Here, CD emerges at moderate carrier densities when singlet and triplet trions (or valley-resolved attractive polarons) preferentially form in opposite valleys (see Fig. 1E, inset). We analyze their valley-dependent oscillator strengths to quantify the spin polarization (see the Supplementary Materials). Because the valley-dependent charge density correlates with the oscillator strength of the transition (*32*, *33*), we estimate that 90% (10%) of charges reside in the valley cross(co)–polarized with the pump even at 8-μm pump-probe separation. This corresponds to a spin polarization *P*_s_ = 0.77, where *P*_s_ *=* (*A*^+^ *− A*^−^)*/*(*A^+^ + A^−^*) and *A^+,−^* is the probe polarization–selective oscillator strength of the trion state under optical pumping. For an electron-doping density of *n* ≈ 1.8 × 10^12^ cm^−2^ (see the Supplementary Materials), this yields an estimated spin population imbalance of ~1.4 × 10^4^ μm^−2^.

**Fig. 2.**
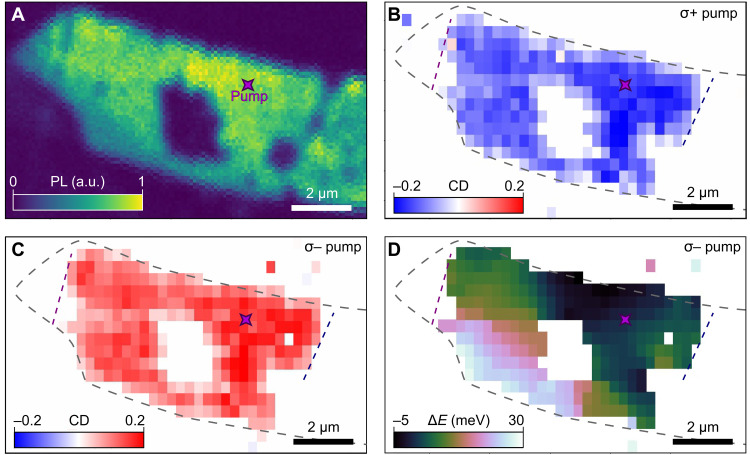
Spatial profile of the spin polarization. (**A**) Sample D1: PL map of the ROI. (**B** and **C**) Map of the CD amplitude across the whole ROI under σ^+^ (B) and σ^−^ (C) pumping located at star in (A). (**D**) Map of peak energy shifts of the singlet CD signal compared to the value at the pump location. Purple and blue dashed lines correspond to wrinkles on the sample. Note that *T* = 4 K, pump power is 7.8 μW, and gate voltage is 0.5 V (*n_e_* ~ 2 × 10^12^ cm^−2^).

The optical pump generates a near-complete free-carrier spin polarization that persists micrometers away from the pump location. We next study this spatial dependence in more detail. Figure 2A shows a PL map of the region of interest (ROI) of the monolayer flake. The central dark area corresponds to a bilayer region. Mapping the CD signal across the entirety of the ROI, Fig. 2B depicts the CD associated with the singlet trion peak as the probe is scanned across the flake while the σ^+^ pump remains fixed. CD is observed within the pristine portion of the ROI, except for in the bilayer region where no resonance peak nor CD signal is found. When the sign of the pump polarization is flipped to σ^−^ (Fig. 2C), the CD signal inverts everywhere. The spatial inhomogeneity of the sample is depicted in Fig. 2D. The trion resonance energy varies by up to 30 meV within the ROI, which is typical even in high-quality heterostructures (*34*), while the CD signal is still robust. However, more prominent imperfections apparently destroy the spin polarization. The purple and blue dashed lines indicate wrinkles and residue in the heterostructure observed under microscope imaging, which correspond to observable dips in PL (Fig. 2A). The CD signal terminates upon crossing the noted defects.

**Fig. 3.**
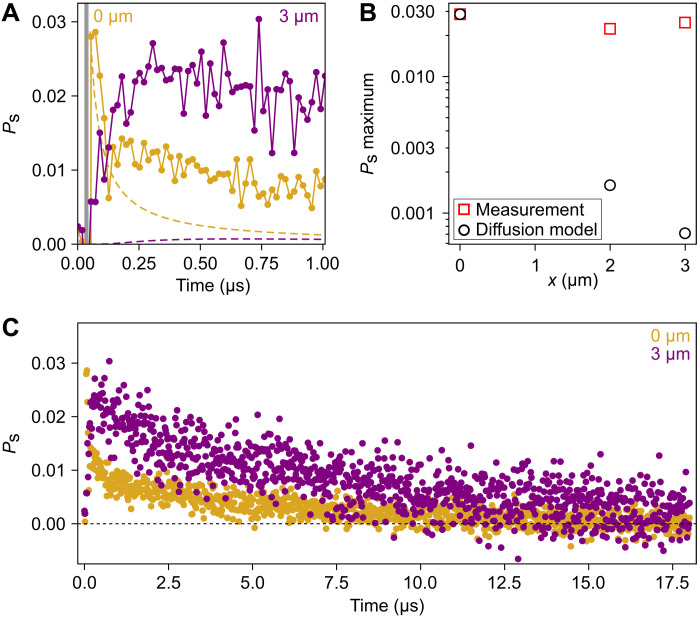
Spin polarization dynamics. (**A**) Sample D2: Time-resolved spin polarization extracted from changes in reflection-probed overlapping (yellow) and 3 μm (purple) from a pulsed circular pump with average power of 2 nW at a repetition rate of 50 kHz. Gray bar corresponds to the window of the pulsed pump. Dashed lines indicate the predicted profiles of a diffusion-decay model. (**B**) Comparison of the maximum spin polarization as a function of pump-probe offset between the measured values and the simulated values of the diffusion-decay model. (**C**) Extended dynamics of the time-resolved spin polarization. Note that *T* = 4 K and gate voltage is 0.5 V (*n_e_* ~ 2 × 10^12^ cm^−2^).

We also investigate the temporal dynamics of the spin polarization with different pump/probe separations (on a second sample D2). We use a local, 5-ns pulsed laser at 633 nm to generate a spin imbalance under the pump and then measure spatially and temporally resolved changes to the spin polarization extracted from differential reflection (see the Supplementary Materials). We observe that as the system evolves in time, mesoscopic spin polarization emerges at micrometer-length scales over a microsecond time scale (Fig. 3A). The peak spin polarization observed exhibits no systematic change with increasing pump/probe separation (Fig. 3B, red squares), agreeing with the spatially uniform CD under CW pumping (Fig. 2). The spin polarization at the pump location sets an upper bound for the optical spin polarization injection. After building up for hundreds of nanoseconds, the spin polarization detected away from the pump location exceeds that observed at the pump location. These results cannot be captured by a 2D diffusion-decay model with pulsed excitation, which predicts at least an order of magnitude decay in polarization at 3-μm separation regardless of the parameter estimates used for the carrier diffusion constant and relaxation time (see the Supplementary Materials) (*35*). These results indicate that after the spin injection from the initial excitation pulse, the spin polarization is appreciably amplified across the sample and persists for more than 10 μs (Fig. 3C).

**Fig. 4.**
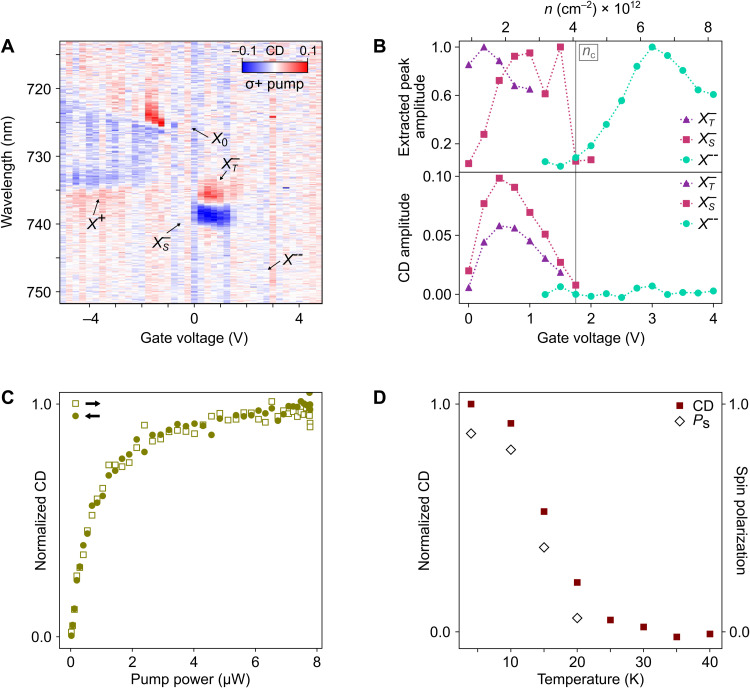
Gate, power, and temperature dependence of the spin polarization. (**A**) Sample D1: Gate-dependent CD spectra probed 8 μm from the pump (Fig. 1B) under σ^+^ pumping. Excitonic states are labeled corresponding to the features in reflection spectra. (**B**) Peak amplitude and CD amplitude extracted from (A) versus gate voltage (doping level). The critical electron density *n_c_* is indicated. (**C**) Sample D1: Power dependence of CD amplitude with pump-probe separation of 1.6 μm and gate voltage of 0.5 V (*n_e_* ~ 2 × 10^12^ cm^−2^). Hollow squares (solid circles) correspond to increasing (decreasing) pump power. (**D**) Sample D3: Temperature dependence of CD amplitude (red squares) with pump-probe separation of 2.2 μm and gate voltage of 1.2 V (*n_e_* ~ 2.8 × 10^12^ cm^−2^). Corresponding spin polarizations (hollow diamond) are extracted from reflection spectra. Note that unless indicated otherwise, *T* = 4 K and pump power is 7.8 μW.

To gain further insight into the origin of the mesoscopic spin order, we vary the doping concentration. We observe that the macroscopic spin polarization strongly depends on electron density and vanishes above a threshold *n_c_* ≈ 4 × 10^12^ cm^−2^. As depicted in Fig. 1C, we can access the intrinsic, hole-doped and highly electron-doped region by varying the gate voltage. Here, we study the long-range CD (i.e., pump-probe separation of 8 μm; Fig. 1B) within these different doping regions, where excitonic resonances are sensitive to underlying spin polarization (*7*, *18*, *19*, *29*–*31*). As shown in Fig. 4A, no CD signal is observed in the intrinsic region, where the neutral exciton resonance is observed in reflection, implying that the optically induced CD is correlated with free carriers in the system. As in Fig. 1F, we observe strong CD co(cross)-polarized to the pump from the triplet (singlet) features. At higher doping concentrations, while the heavily doped charged exciton *X*^--^ (*31*) is observed in reflectivity, there is no observable CD. We further characterize this by plotting the extracted peak and CD amplitudes (Fig. 4B) against the estimated doping density for different species of excitonic states. We observe that at 1.75 V, *n_c_* ≈ 4 × 10^12^ cm^−2^, where the Fermi energy is still within the spin-orbit gap in the conduction band (*29*), the system transitions from observable singlet and triplet trions with strong CD to *X*^--^ exciton states with no observable CD. As the *X*^--^ exciton states are sensitive to spin polarization of the free carriers (*7*, *29*, *31*), the disappearance of CD in this doping regime indicates the free carriers are unpolarized, agreeing with the previously reported first-order phase transitions in MoS_2_ (*8*). We also observe CD signal from the positive trion X^+^ in the hole-doped regime.

Last, we investigate the spin polarization with respect to changes in pumping power and temperature (on a third sample D3). Long-range spin polarization is generated with submicrowatt pumping power and saturates quickly without displaying hysteresis effects (Fig. 4C). Figure 4D depicts the temperature dependence of the CD signal and calculated spin polarization. The CD signal vanishes at *T* = 30 K, although reflection spectra still exhibit clear resonance features of singlet/triplet trions and X^--^ states (see the Supplementary Materials). The rapid transition from an unpolarized (*P*_s_ = 0.06) to a polarized (*P*_s_ = 0.87) spin state as the temperature goes below *T* = 15 K evidences a phase transition.

The key finding of this work is the optical generation of mesoscopic spin polarization in a TMD monolayer. A possible explanation for long-range spin polarization is diffusion of optically pumped resident carriers. In such a noninteracting spin diffusion picture, the spatial spread of an initial, conserved spin imbalance would yield a rapid decay in the maximum spin polarization away from the pump region. Our time-resolved measurements show a uniform maximum spin polarization micrometers away from the location of the pulsed pump. The corresponding excess of polarized spins—40 times larger than predicted by diffusion—necessitates interaction-driven amplification. In addition, the complete disappearance of long-range spin polarization with small changes in carrier density is inconsistent with noninteracting models. Furthermore, in a diffusion model, the reduction of spin lifetime with increased doping or temperature (*19*) could eventually quench the spin polarization, leading to shorter diffusion lengths and a spatial contraction in the CD profile. This spatial contraction predicted by the diffusion model is not observed (see the Supplementary Materials). In both the CW and pulsed pump measurements, the observed spin polarization behavior requires electron-electron interactions absent from a diffusion model.

However, our results show excellent agreement with recent theoretical and experimental work on magnetism in monolayer TMDs. In theoretical models of electron-electron interactions in TMDs (*10*–*16*), exchange inter- and intravalley coupling lead to spin-polarized ferromagnetic phases at electron densities around *n_e_ ~* 10^12^ cm^−2^. These models were validated by experimental studies of magnetic phases in electron-doped monolayer molybdenum disulfide (MoS_2_) in an external magnetic field. In that case, strong exchange intervalley interactions—compared to the small spin-orbit splitting of the conduction bands in MoS_2_—lead to band inversion and the spin polarization of resident electrons across both K and K′ valleys (i.e., spin-polarized but not valley-polarized electrons) (*7*, *13*). In contrast, WSe_2_ monolayers exhibit an order of magnitude larger spin-orbit splitting in the conduction band (*12*). The Fermi level in the electron-doped regime remains within this spin-orbit gap *(**29*), implying that only the bottom two conduction bands are occupied by resident electrons. Consequently, the predicted ferromagnetic ground state consists of spin-/valley-polarized resident electrons (*12*), in agreement with our CD results. Moreover, the sudden disappearance of spin polarization with increased electron doping is consistent with a phase transition from a ferromagnetic to a paramagnetic phase, as found in previous experimental (*8*) and theoretical studies (*10*–*16*) of magnetic phase transitions in TMDs. This phase transition was interpreted to be first order (*8*). As in these previous studies (*7*, *8*), the magnetic phase transition in our data is accompanied by a sudden transition from trion states to X^--^ states. While the nature of this excitonic transition remains an ongoing topic of research—having been associated with abrupt changes in the internal correlations of the exitonic states (*31*) and in the electron effective mass (*8*)—its sharpness suggests a first-order transition. However, the CD magnitude does not show a distinct discontinuity, making it challenging to definitively conclude the order of the magnetic phase transition. Recent theory work has found that the transition may be first or second order, depending on the interaction strength (*15*). A transition also is observed in the temperature dependence of the CD signal, which represents the magnetization (*36*), displaying a trend qualitatively similar to other 2D ferromagnetic materials (*37*). Criticality fits indicate a Curie temperature *T_C_* = 15 K with a critical exponent of 0.113, close to the value of 0.125 for a 2D Ising model (see the Supplementary Materials) (*38*). The consistency of our results with these ferromagnetic models suggests that the mesoscopic spin polarization is associated with ferromagnetic order in the monolayer TMD.

An exact microscopic description of the ferromagnetic state and its optical control remain topics of ongoing interest. Current proposals picture a ground state of fluctuating, nanometer-scale ferromagnetic domains, which, averaged over space and time, show no net magnetization (*8*, *15*). The circularly polarized optical pump stabilizes the magnetic state against fluctuations by selectively valley-pumping spin-polarized electrons, thereby breaking the symmetry between degenerate magnetic states and preferentially favoring the formation of a copolarized magnetic state. This mechanism is also fundamentally different from previously reported all-optical control of magnetism, which is based on heating and inverse Faraday effects (*39*–*42*). Further, while the optical pump acts locally, the magnetic order is stabilized mesoscopically, extending well beyond the submicrometer pumping region to the boundaries of the monolayer. Recent theoretical works have argued that spin injection into an unstable, symmetric ferromagnetic state can yield spin amplification, similar to our observations (*43*). Continued theoretical and experimental study is needed to better understand the microscopic nature of this itinerant ferromagnetism and its stabilization by light.

## DISCUSSION

We present evidence for optically controllable ferromagnetic order in TMDs. The recent discovery of magnetic 2D materials has generated excitement because of their novel integration and heterostructure possibilities (*44*–*46*). Our research establishes TMDs as a 2D magnetic material, albeit with very different physics and properties than more conventional 2D magnets—the magnetism originates from strongly correlated itinerant electrons, and critically, the magnetic configuration can be fully tuned nonlocally with optical fields and electronic gating. Moreover, the local optical pump stabilizes the magnetic state, even at low submicrowatt power, providing finer spatial resolution for the study and control of magnetic domain structure. These unique features open new avenues for probing the previously inaccessible physics of magnetic order in 2D semiconductors, prompting future experimental investigations of the temporal dynamics with ultrafast spectroscopy (*47*) and spatial formation of domains with nitrogen-vacancy center in diamond magnetic sensors (*48*–*50*) and Lorentz transmission electron microscopy (*51*) under optical pumping. In addition, TMDs are a prototypical platform for explorations of correlated phenomena in 2DEGs, and we show that optical pumps provide a powerful tool for understanding and controlling these systems. For instance, magnetic phases and their CD could be used to manipulate and probe Mott insulators and Wigner crystals (*3*–*5*).

Furthermore, our findings will accelerate technological developments using TMDs, already a leading material platform for investigating next-generation spin, valley, and optoelectronics (*52*–*55*). Specifically, the discovery of optically reconfigurable magnetism and CD in atomically thin semiconductors will stimulate the design of nonreciprocal optoelectronics and photonics (*56*–*58*), such as on-chip all-optical isolators with built-in optical memory. In addition, spin amplification enables fan out, a necessary element in envisioned spintronic circuits (*43*). Our research indicates that magnetic phases in TMDs can fulfill this amplification criteria. Last, our research creates a bridge between magnetism and optical control in TMDs, which can be leveraged for direct interfacing between integrated photonics and magnetic solid-state memories (*42*), suggesting new routes for neuromorphic optical computing (*59*).

## MATERIALS AND METHODS

### Sample fabrication

The monolayer tungsten diselenide (WSe_2_), hBN, and FLG flakes are mechanically exfoliated from commercial bulk crystal (WSe_2_-2D semiconductor; hBN and FLG, HQGraphene) onto Si/SiO_2_ chips. The thickness and cleanliness of the flakes are first examined with optical microscopy and then by atomic force microscopy (fig. S1A). We used an all-dry transfer method 1 to fabricate the hBN-encapsulated WSe_2_ stack with the FLG top gate and contact. Electrodes are patterned via photolithography and then deposited by electron beam physical deposition with 5-nm Ti and 95-nm Au (fig. S1B). We also note that sample D2 has a layer of poly(methyl methacrylate) on top of the completed heterostructure.

### Optical measurement setup

The samples were kept in a closed loop cryostat (Montana Instruments) at 4 K during the experiment unless otherwise claimed. The optical setup is depicted in fig. S2. The two galvo mirrors control the pump and probe beam independently to realize spatial scans. We use a 660-nm diode laser (Thorlabs) as the optical pump exciting through port 3 with a bandpass filter to spectrally clean the pump laser. A supercontinuum laser (YSL Photonics) is deployed as the broadband probe sent through port 1. The reflection of the supercontinuum from the sample is collected by port 2 and fiber coupled to a spectrometer with charge-coupled device (Teledyne Princeton Instruments) to realize spectrally resolved reflection measurements. The polarizations of each beam are independently controlled by linear polarizers and half-wave plates to allow different pump/probe polarization combinations.

To map the PL from the sample (Fig. 2A), we use ports 1 and 2 as the pump and collection channels, respectively. The same 660-nm diode laser is used for pumping. The PL signal is collected from port 2 and detected by an avalanche photodiode (Excelitas). By scanning galvo mirror 1, the colocalized pump and collection are simultaneously moved across the sample, realizing the PL mapping.

In time-resolved measurements, we use a 633-nm pulsed diode laser with pulse duration of 5 ns and repetition rate of 50 kHz as the pump at port 3 and a tunable CW Ti:sapphire laser as the probe at port 1 with subnanowatt power. The probe is tuned to the triplet trion resonance, and the reflection is collected at port 2 with an APD (Excelitas). Time correlation between the probe and pump is measured by a time tagger (Qutag).

## Supplementary Materials

### This PDF file includes:

Supplementary Text Figs. S1 to S11 Table S1 References
