# Supplementary material for: Optically controllable magnetism in atomically thin semiconductors
Source: Sci Adv. 2022 Sep 30;8(39):eabq7650. doi: 10.1126/sciadv.abq7650 (PMC9524837; doi:10.1126/sciadv.abq7650)
Supplement: Supplementary file 1 — Supplementary Text Figs. S1 to S11 Table S1 References [file sciadv.abq7650_sm.pdf]

Science Advances
AAAS

Supplementary Materials for

Optically controllable magnetism in atomically thin semiconductors

Kai Hao *et al.*

Corresponding author: Alexander A. High, ahigh@uchicago.edu

Sci. Adv. **8**, eabq7650 (2022)
DOI: 10.1126/sciadv.abq7650

This PDF file includes:

Supplementary Text
Figs. S1 to S11
Table S1
References

Supplementary Text

Capacitor model of charge density estimation

To estimate the charge carrier density under electrostatic gating, we model the heterostructure as a parallel plate capacitor (*7*): the FLG and monolayer WSe_2 flakes are the electrodes separated by the dielectric top hBN flake. The capacitance per unit area is given by $C = \frac{\epsilon_{hBN}\,\epsilon_0}{d_{hBN}}$. Using the relative permittivity of hBN $\epsilon_{hBN} = 3.76$ (*60*) and the hBN thickness $d_{hBN} = 11.5$ nm, we find $C \approx 290$ nF cm^{-2}. The carrier density as a function of gate voltage is given by $n(V_g) = C\,(V_g - V_0)$, where V_0 is the gate voltage corresponding to the intrinsic regime with no carrier doping. By comparison with the neutral exciton feature in reflection (Fig. 1c), we approximate $V_0 = -0.5$ V.

Line shape analysis

To estimate the valley/spin polarization of the free carriers, we extract the total oscillator strength of the trions in the two valleys. By comparing the oscillator strengths, we approximate the relative density of states in each valley, which give an estimation of the polarization of the free carriers (*32,33*).

The reflection contrast corresponding to the two trion features from Fig. 1e is depicted in Fig. S3, which shows an asymmetric Fano-like line shape. The reflection contrast is given by $RC = \frac{R}{R_{ref}} - 1$, where R is the bare reflectivity (Fig. 1e) and R_{ref} is a reference reflectivity where the trion oscillator strengths disappear, under large gate bias or in the intrinsic region (*18*). Such spectra can be captured by a two-peak Breit-Wigner-Fano (BWF) line shape fitting:

$$I(\omega) = I_1 \frac{(1 + \frac{\omega - \omega_1}{q_1 \Gamma_1})^2}{1 + (\frac{\omega - \omega_1}{\Gamma_1})^2} + I_2 \frac{(1 + \frac{\omega - \omega_2}{q_2 \Gamma_2})^2}{1 + (\frac{\omega - \omega_2}{\Gamma_2})^2} + C \quad (1)$$

where $q_{1/2}$ is the parameter which captures the asymmetry of the line shape. When $\frac{1}{q} \rightarrow 0$, the function converges to the Lorentzian line shape, where $I_{1/2}$ is the amplitude, $\omega_{1/2}$ is the center energy, and $\Gamma_{1/2}$ is the linewidth (*61,62*).

By fitting the RC to a superposition of BWF line shapes with two resonances corresponding to the singlet and triplet trions (Fig. S3 lines), we extract the fitting parameters shown in table T1. The oscillator strength of each resonance is proportional to the area of the Lorentzian line

shape $A = I_0 \Gamma$. By comparing the relative oscillator strengths for each state under co- and cross-circular pumping, we can estimate the ratio of the density of states in each valley. We take this ratio, 90:10 for the triplet trion, as the ratio between the valley-polarized spin states of the resident electrons. By similarly fitting pump-off RC spectra, balanced detection gives a polarization sensitivity as low as 3%.

Sample D2 characterization and comparison of temporal dynamics with diffusion

The temporal dynamics are measured on a different sample D2. Characteristic reflection spectra and CD spectra are shown in Figure S4, which show similar features as sample D1 in main text Fig. 1. Using the time-resolved, circularly polarized differential reflection measurement results at selected positions with different distances from the pump, we compare the temporal profile with that predicted in a diffusion picture. Here the analysis is performed on an additional dataset taken from different pump and probe positions than presented in main text Fig. 3.

The spin polarization under pulsed pumping is extracted from the differential reflection in the CW pumping case:

$$P_s^{pulse}(t) = \frac{\Delta R^{pulse}(\lambda, t)}{\Delta R^{CW}(\lambda)} P_s^{CW} \quad (2)$$

P_s^{CW} is the spin polarization found from the line shape analysis of the oscillator strength transfer under CW pumping. $\Delta R^{pulse/CW}$ is the differential reflection at wavelength of interest λ under pulsed/CW pumping. A clear build-up process is measured as a rising edge in the time resolved measurements as shown in Figure S5a for a pump-probe separation of 4.87 μm. Thus, we tentatively fit the temporal profile with a two-dimensional diffusion-decay model (*35*)*:*

$$P_s(x, t) = \frac{\sigma_0^2 \, P_s^0}{\sigma_0^2 + 4Dt} e^{-x^2/(\sigma_0^2+4Dt)} e^{-t/T} \quad (3)$$

where D is the diffusion constant, T is the spin polarization lifetime, and σ_0 = 0.78 μm is the spatial convolution of the pump and probe beam sizes.

While the fitting cannot accurately capture the full temporal evolution of the spin profile (Fig. S5b), the diffusion constant extracted by fitting the rising edge (Fig. S5a) provides an estimate of ~ 0.065 $cm^2 \, s^{-1}$ for the propagation speed of the spin order formation. This value is significantly lower than the measured diffusion constant in previous research (*35*), indicating a

different mechanism behind the spin order propagation. The decay trend gives a spin lifetime of ~ 52 μs (Fig. S5b).

Following the analysis in the main text (Fig. 3), we compare the measured temporal profiles for various pump-probe separations with those of the diffusion model (Fig. S6). Ignoring decay, diffusive transport of a fixed number of spins away from a local source should lead to an approximately $1/r^2$ dependence in the peak polarization measured a distance r from the source. This geometric constraint suggests a rapid fall off in the maximum spin polarization away from the pump, as seen in Fig. S6b for a diffusion model with representative D = 0.035 cm^2 s^{-1} and infinite lifetime. In contrast, the measured maximum polarization shows no systematic change as the offset varies, even increasing at larger distances (Fig. S6a). As in Fig. 3, the measured spin polarization is amplified by almost an order of magnitude compared to purely diffusive propagation (Fig. S6c). The variations in maximum polarization can be attributed to local deviations in the dielectric environment or defects distributed across the sample (*34*). The strength of the electron-electron interactions, which creates the ferromagnetic state, will vary throughout the sample due to these inhomogeneities. Correspondingly, the maximum spin polarization will also vary.

Hysteresis check

In addition to sweeping the pump power (Fig. 4c), we test magnetic hysteresis in D1 by continuously varying the pump polarization in opposite directions. No observable hysteresis loop is observed (Fig. S7). Similarly, previous measurements in monolayer MoS_2 do not observe hysteresis under an applied magnetic field (*7*).

Sample D3 characterization and temperature dependence

The temperature-dependent measurements (Fig. S8) are performed on a third sample (D3). Figure S8a shows gate-dependent reflection spectra at 4 K with a pump-probe separation of 2.2 μm, which display similar doping regimes as sample D1 (Fig. 1c). In the electron-doped region, strong CD signal is observed. Figure S8b depicts the reflection and CD spectra at 30 K. While the electron-doped CD vanishes at this elevated temperature, the singlet and triplet trion features remain, confirming that the temperature dependence of the CD signal probes that of the spin polarization. Temperature dependent data was also taken with overlapped pump and probe. The

CD amplitude shows a very similar trend as that with a 2.2 μm pump-probe offset (Fig. S8c). The criticality fit $\alpha\ (T_c - T)^{\beta}$ is applied to the CD data (*37*), extracting a critical temperature $T_c = 15.0\ K\ (15.3\ K)$ and a critical exponent $\beta =\ 0.113\ (0.106)$ for the offset (overlapping) pump-probe configuration. For the 2D Ising model, the expected critical exponent is $\beta =\ 0.125$.

<u>Sample D4 characterization and comparison of temperature- and gate-dependent results with steady-state diffusion</u>

Here, we test predictions of the spin diffusion model under CW pumping against additional experimental data taken from a fourth sample (D4) (Fig. S9).

1. Temperature and doping dependence of the CD spatial profile

In previous research (*19*), the spin lifetime of electrons is strongly dependent on the temperature and doping level. In a simple diffusion model the spin diffusion length is directly correlated with the spin lifetime. Thus, within the diffusion model, the spatial profile of the CD signal would have a strong temperature and doping dependence. In Figure S10, the spatial profiles of the CD signal at varying temperatures and doping levels are shown. The CD profiles uniformly decrease by an order of magnitude with no observable spatial contraction at elevated temperatures or increased doping. Thus, our observation is in stark contrast to the predictions of the diffusion model.

2. Power saturation curves under different temperatures

In a spin pumping model with a CW laser, the steady state spin polarization results from competition between the spin pumping rate and the spin decay rate. Within this model, if the spin decay rate increases at increasing temperature, the loss of spin polarization could be compensated by increasing the pumping rate. This can be understood with simple rate equations for a single pump in the K valley:

$$\frac{dN_+}{dt} = -GN_+ - \gamma(N_+ - N_-) \quad (4)$$

$$\frac{dN_-}{dt} = GN_+ - \gamma(N_- - N_+) \quad (5)$$

Where $N_{+(-)}$ are the number of electrons in the K (K') valley, G is the spin pumping rate which is linear to the pumping power, and γ is the intervalley scattering (spin decay) rate. The steady state under CW pumping will have a spin polarization of:

$$\frac{N_+ - N_-}{N_+ + N_-} = -\frac{1}{1 + \frac{2\gamma}{G}} \quad (6)$$

Within this model, the spin polarization should always saturate at a value of 1, as increases in the spin decay rate can always be offset by increasing the pump power – in other words, the saturated spin polarization should be largely temperature independent. To test this prediction, we measure power-dependent CD signal under different temperatures. As depicted in Fig. S11a, the CD signal saturates at similar powers, while the saturated CD amplitude decreases by an order of magnitude. The photoluminescence (PL) power dependence (Fig. S11b) indicates that the pumping is within the linear absorption regime, meaning the simple rate model should still hold. The saturated CD, then, is solely determined by the temperature. This directly contradicts the predictions of a simple spin pumping model. Such phenomena, on the other hand, is predicted by the temperature curve with a 2D magnetic order as discussed in the main text.

To summarize, a spin diffusion picture predicts dramatically decreasing of spin polarization under pulse pumping away from the pump and the spatial distribution should strongly depend on temperature and doping level. In contrast, our measurements show spin polarization significantly larger than the prediction, and with no observable temperature and doping dependence of the spatial profile, only the magnitude of the CD. Also, the power dependent CD amplitude curve under different temperatures significantly deviates from the prediction of the spin pumping/diffusion picture. Given the inadequacy of such a spin pumping/diffusion model to explain the complete dataset, magnetic interactions must account for the observation of long-range spin polarization generated by an optical pump.

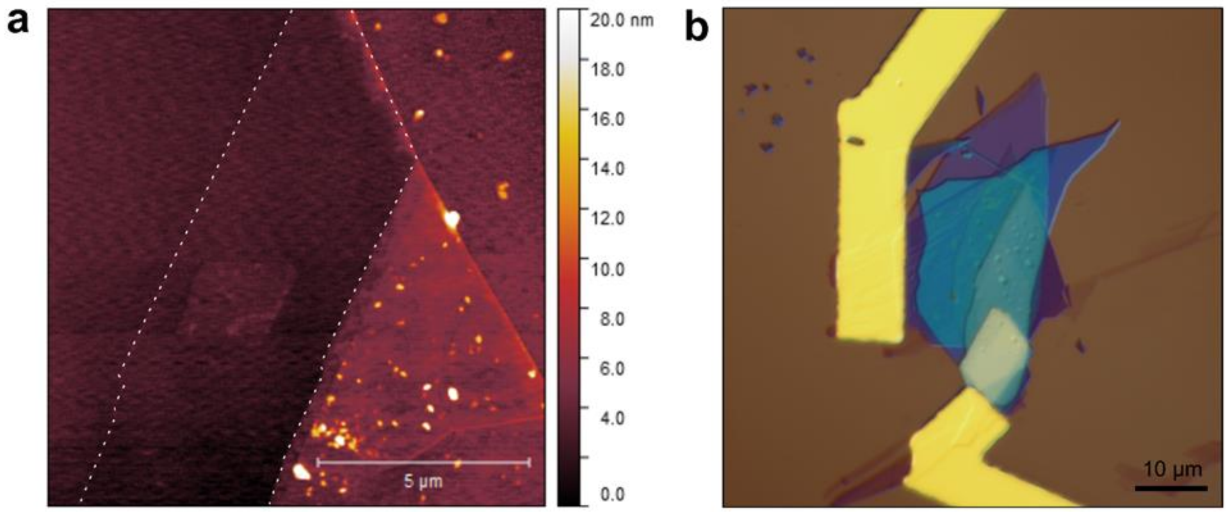

Fig. S1. Sample fabrication.

a, AFM image of the WSe_2 flake on Si/SiO_2 chip with monolayer region outlined. **b,** Optical microscope image of the full heterostructure D1 with patterned contacts.

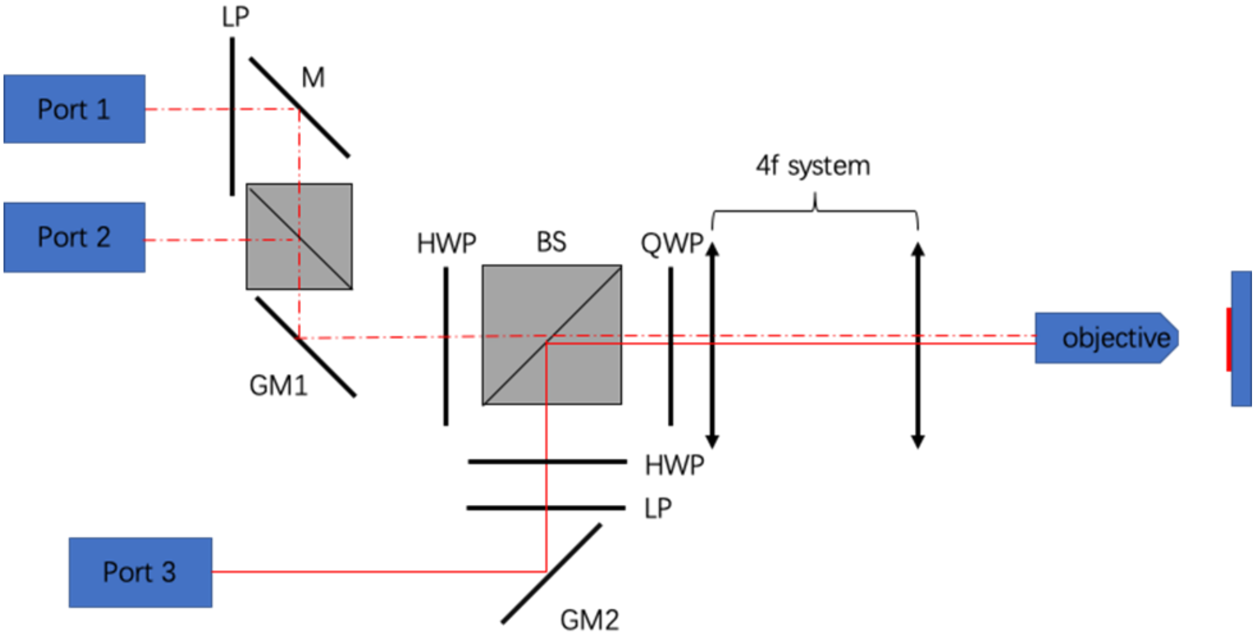

Fig. S2. Optical setup.

Setup components are: Port – fiber launcher, M – mirror, GM – Galvo mirror, LP – linear polarizer, HWP – half-wave plate, QWP – quarter-wave plate, BS – beam splitter, 4f system – lens pair, objective – NA = 0.75.

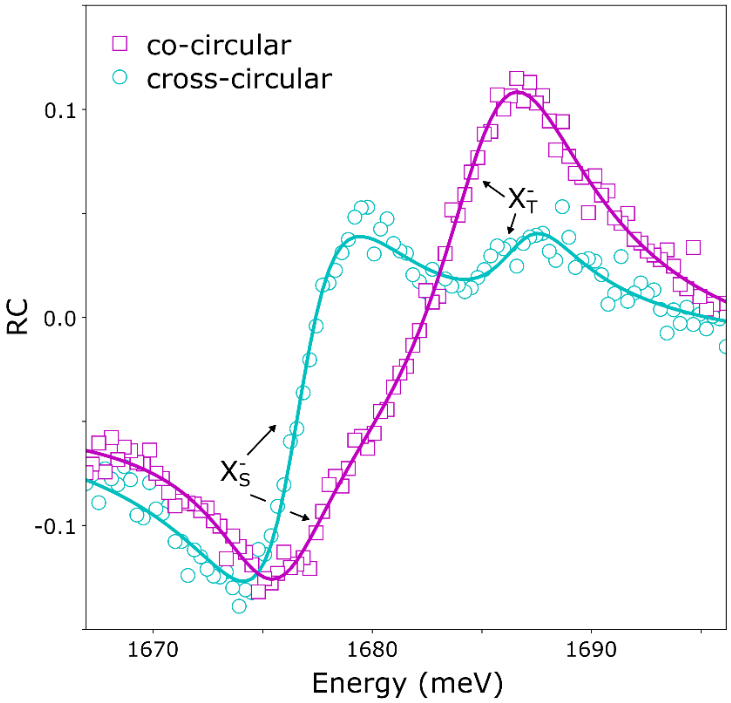

Fig. S3. Line shape fitting.

Polarized reflection contrast spectra showing singlet (X_S^-) and triplet (X_T^-) trion features under optical pumping. The pink (blue) dots correspond to the probe being polarized co- (cross-) circular to the pump. BWF fittings are plotted. Note: T = 4 K, pump power is 7.8 μW, and pump-probe offset is 8 μm.

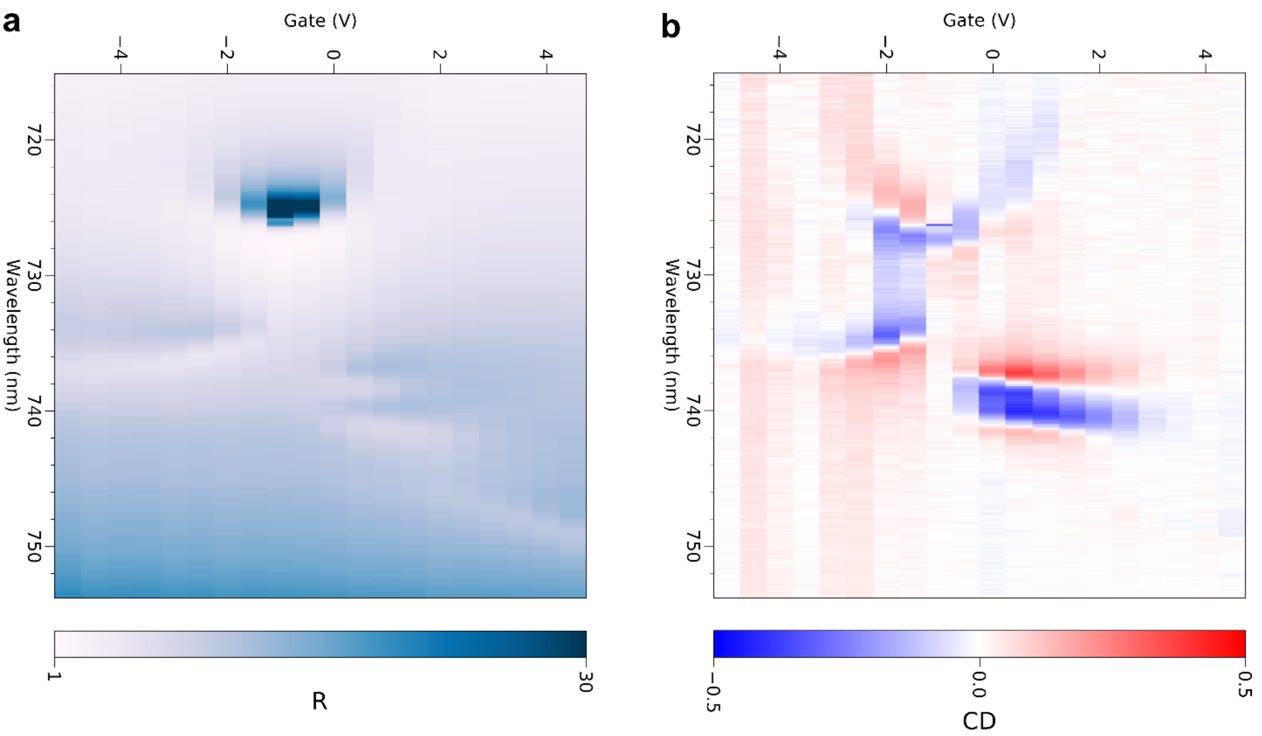

Fig. S4. Sample D2 characterization.

Gate-dependent reflection (**a**) and CD spectra (**b**) at 4 K with a pump-probe separation of 3 μm and pump power of 7.8 μW.

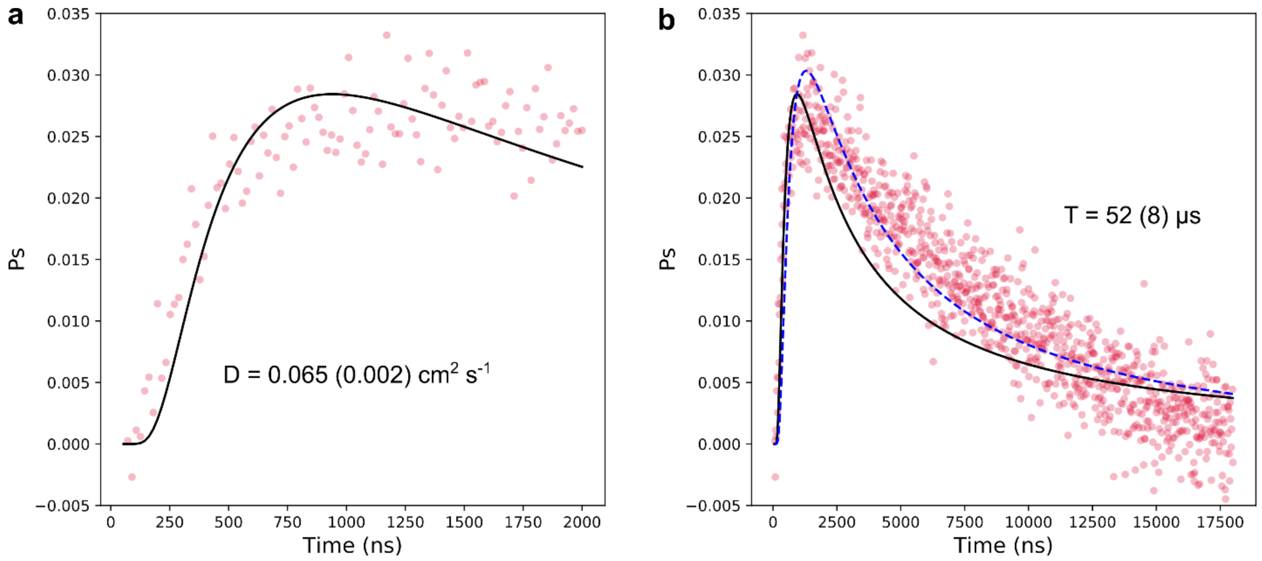

Fig. S5. Time resolved measurement for point 4.78 μm away from pump.

(**a**) Sample D2: Rising edge profile with two-dimensional diffusion-decay model fitting (black line). (**b**) Fitting the full-time range with D = 0.065 cm^2 s^{-1} from the rising edge (black solid line) fitting yields lifetimes beyond a second (*i.e.*, no decay). Fitting both D and T (blue dashed line) yields D = 0.045 cm^2 s^{-1} and T = 52 μs. Fitting uncertainties are in parentheses. Note: T = 4 K, average pump power is 2 nW at a repetition rate of 50 kHz, and gate voltage is 0.5 V (n_e $\sim 2 \times 10^{12}\ cm^{-2}$).

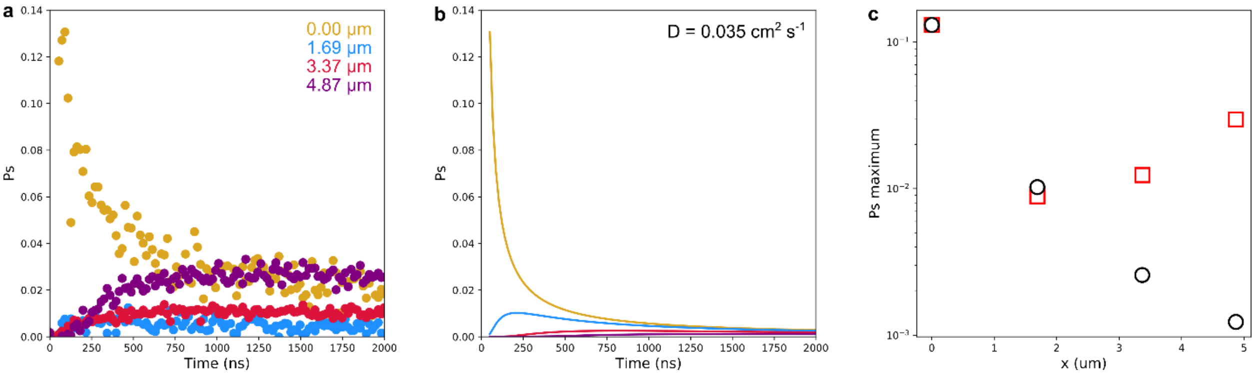

Fig. S6. Additional time resolved data.

(**a**) Sample D2: Time resolved measurement for different pump-probe separations. (**b**) Simulation of diffusion model with diffusion constant D = 0.035 cm^2 s^{-1} for different separations. (**c**) Comparison between the measured (red squares) and simulated (black circles) maximum spin polarization. Note: T = 4 K, average pump power is 2 nW at a repetition rate of 50 kHz, and gate voltage is 0.5 V (n_e ~$2 \times 10^{12}\ cm^{-2}$).

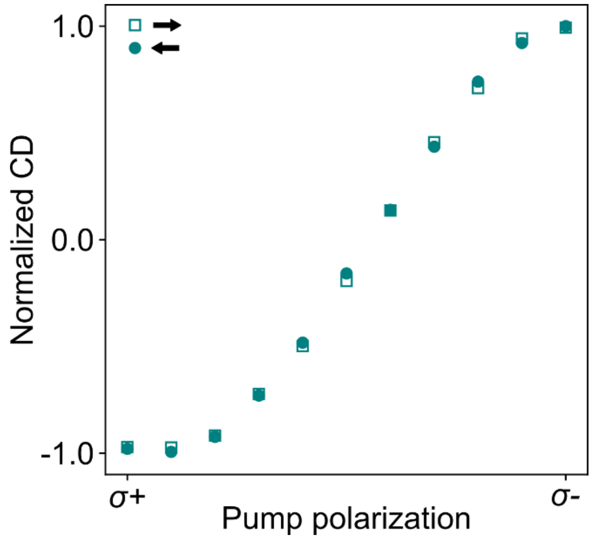

Fig. S7. Pump polarization sweep.

Sample D1: Polarization dependence of singlet CD amplitude. Hollow squares (solid circles) correspond to sweeping the polarization from σ+ to σ- (σ- to σ+). Note: T = 4 K, pump power is 7.8 μW, pump-probe offset is 1.6 μm, and gate voltage is 0.5 V ($n_e \sim 2 \times 10^{12}\ cm^{-2}$).

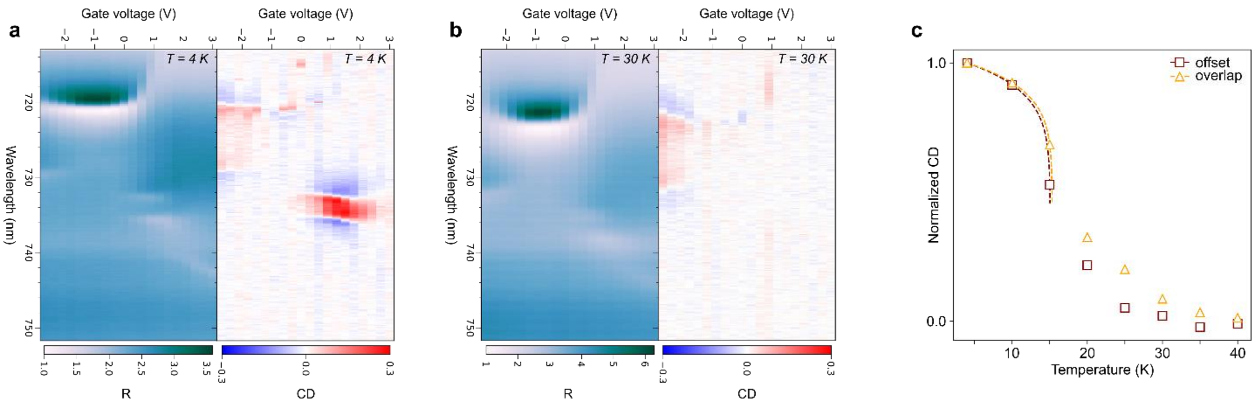

Fig. S8. Sample D3 characterization and temperature dependence.

a, Gate-dependent reflection (left) and CD (right) spectra at 4 K with a pump-probe separation of 2.2 µm. **b,** Gate-dependent reflection (left) and CD (right) spectra at 30 K. **c,** Temperature dependence of triplet CD amplitude selected from gate-dependent CD spectra at gate voltage of 1.2 V ($n_e \sim 2.8 \times 10^{12}\ cm^{-2}$). Hollow squares (triangles) correspond to experimental data for offset (overlapping) pump and probe with respective fittings (dashed lines). Note: Pump power is 7.8 µW.

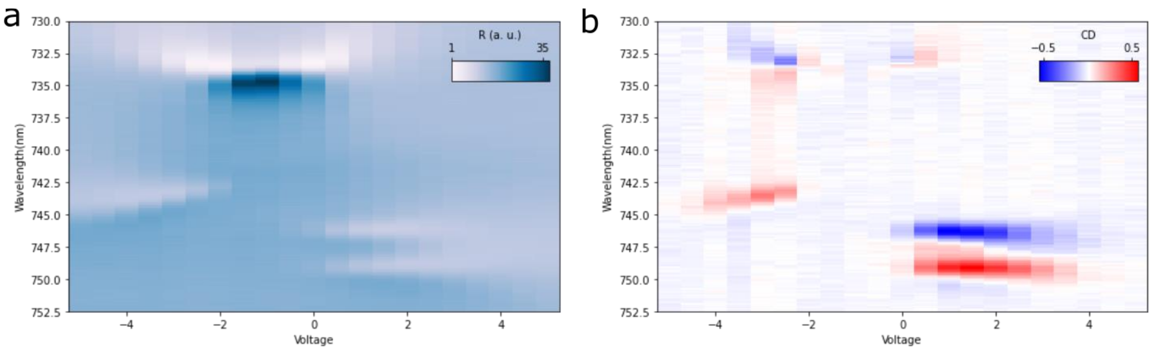

Fig. S9. Sample D4 characterization.

Gate-dependent reflection (**a**) and CD spectra (**b**) at 4 K with a pump-probe separation of 2.5 µm and pump power of 7.8 µW.

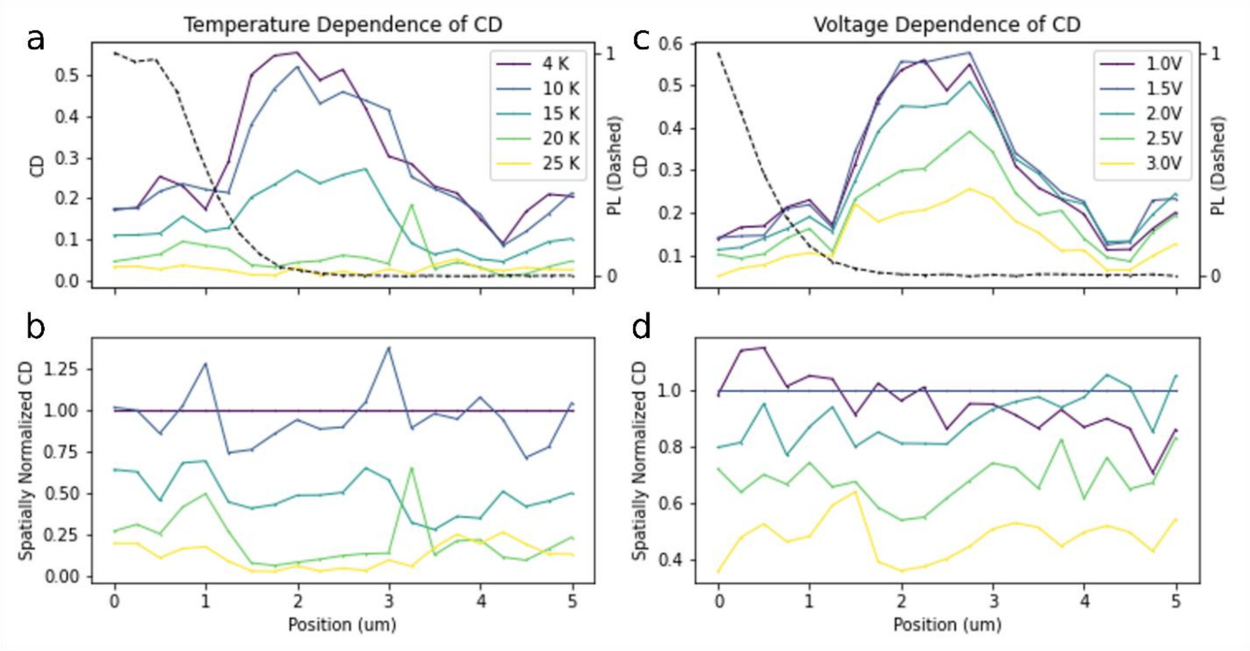

Fig. S10. Temperature and gate dependence of CD spatial profiles.

Sample D4: Measured (**a**) and normalized (to 4 K) (**b**) CD spatial profiles observed at various temperatures with a fixed gate voltage of 1.5 V (n_e ~$2.8 \times 10^{12}\ cm^{-2}$). Measured (**c**) and normalized (to 1.5 V) (**d**) CD spatial profiles observed at various gate biases (n_e from ~$2 \times 10^{12}\ cm^{-2}$ to ~ $4 \times 10^{12}\ cm^{-2}$) with a fixed temperature of 4 K. Dashed line indicates the PL intensity, as guidance of pumping spot profile. Note: pump power is 7.8 µW.

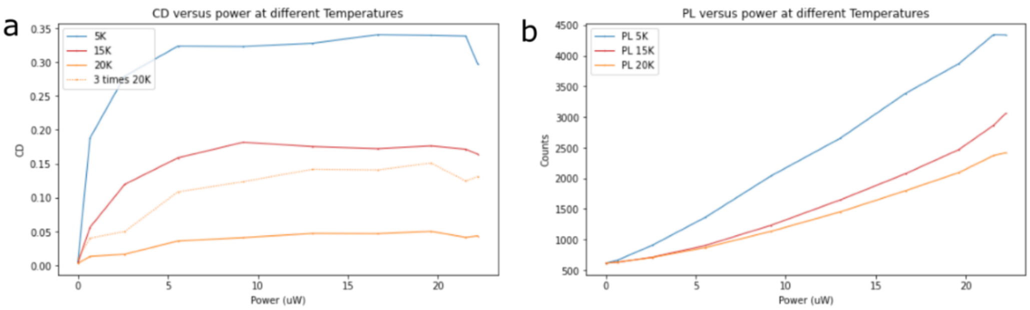

Fig. S11. Power dependence of CD and PL amplitudes under different temperatures. Sample D4: Power-dependent CD with a pump-probe separation of 1.8 µm (**a**) and PL (**b**) amplitudes. Note: Gate voltage is 1.5 V (n_e ~$2.8 \times 10^{12}\ cm^{-2}$), and pump power is 7.8 µW.

	Triplet					Singlet					
	I_1	ω_1 (meV)	Γ_1 (meV)	q_1	$A_1 = I_1\Gamma_1$	I_2	ω_2 (meV)	Γ_2 (meV)	q_2	$A_2 = I_2\Gamma_2$	C
Co-circular	0.151	1685.4	4.1	3.5	0.62	0.0005	1675.8	3.3	0.08	0.0016	-0.13
Cross-circular	0.035	1687.0	2.3	2.8	0.08	0.075	1676.5	2.7	0.9	0.20	-0.13
Ratio	~90:10					~99:1					
P_s	0.77					-0.98					

Table S1. Fitting the trion features under optical pumping.

REFERENCES AND NOTES

1. K. S. Kim, S. A. Kivelson, Discovery of an insulating ferromagnetic phase of electrons in two dimensions. *Proc. Natl. Acad. Sci. U.S.A.* **118**, e2023964118 (2021).

2. M. S. Hossain, M. K. Ma, K. A. V. Rosales, Y. J. Chung, L. N. Pfeiffer, K. W. West, K. W. Baldwin, M. Shayegan, Observation of spontaneous ferromagnetism in a two-dimensional electron system. *Proc. Natl. Acad. Sci. U.S.A.* **117**, 32244–32250 (2020).

3. E. C. Regan, D. Q. Wang, C. H. Jin, M. I. Utama, B. N. Gao, X. Wei, S. H. Zhao, W. Y. Zhao, Z. C. Zhang, K. Yumigeta, M. Blei, J. D. Carlstrom, K. Watanabe, T. Taniguchi, S. Tongay, M. Crommie, A. Zettl, F. Wang, Mott and generalized Wigner crystal states in WSe_2/WS_2 moiré superlattices. *Nature* **579**, 359–363 (2020).

4. Y. Zhou, J. Sung, E. Brutschea, I. Esterlis, Y. Wang, G. Scuri, R. J. Gelly, H. Heo, T. Taniguchi, K. Watanabe, G. Zarand, M. D. Lukin, P. Kim, E. Demler, H. Park, Bilayer Wigner crystals in a transition metal dichalcogenide heterostructure. *Nature* **595**, 48–52 (2021).

5. T. Smolenski, P. E. Dolgirev, K. Clemens, A. Popert, Y. Shimazaki, P. Back, X. B. Lu, M. Kroner, K. Watanabe, T. Taniguchi, I. Esterlis, E. Demler, A. Imamoglu, Signatures of Wigner crystal of electrons in a monolayer semiconductor. *Nature* **595**, 53–57 (2021).

6. F. C. Wu, T. Lovorn, E. Tutuc, A. H. MacDonald, Hubbard model physics in transition metal dichalcogenide moiré bands. *Phys. Rev. Lett.* **121**, 026402 (2018).

7. J. G. Roch, G. Froehlicher, N. Leisgang, P. Makk, K. Watanabe, T. Taniguchi, R. J. Warburton, Spin-polarized electrons in monolayer MoS_2. *Nat. Nanotechnol.* **14**, 432–436 (2019).

8. J. G. Roch, D. Miserev, G. Froehlicher, N. Leisgang, L. Sponfeldner, K. Watanabe, T. Taniguchi, J. Klinovaja, D. Loss, R. J. Warburton, First-order magnetic phase transition of mobile electrons in monolayer MoS_2. *Phys. Rev. Lett.* **124**, 187602 (2020).

9. G. B. Liu, D. Xiao, Y. G. Yao, X. D. Xu, W. Yao, Electronic structures and theoretical modelling of two-dimensional group-VIB transition metal dichalcogenides. *Chem. Soc. Rev.* **44**, 2643–2663 (2015).

10. D. K. Mukherjee, A. Kundu, H. A. Fertig, Spin response and collective modes in simple metal dichalcogenides. *Phys. Rev. B* **98**, 184413 (2018).

11. J. E. H. Braz, B. Amorim, E. V. Castro, Valley-polarized magnetic state in hole-doped monolayers of transition-metal dichalcogenides. *Phys. Rev. B* **98**, 161406(R) (2018).

12. M. Van der Donck, F. M. Peeters, Rich many-body phase diagram of electrons and holes in doped monolayer transition metal dichalcogenides. *Phys. Rev. B* **98**, 115432 (2018).

13. D. Miserev, J. Klinovaja, D. Loss, Exchange intervalley scattering and magnetic phase diagram of transition metal dichalcogenide monolayers. *Phys. Rev. B* **100**, 014428 (2019).

14. D. Miserev, J. Klinovaja, D. Loss, Magnetic phase transitions in two-dimensional two-valley semiconductors with in-plane magnetic field. *Phys. Rev. B* **103**, 024401 (2021).

15. P. Fan, Z. G. Zhu, Magnetic order transition in monolayer MoS_2 induced by strong intervalley correlation. *Phys. Rev. B* **104**, 195417 (2021).

16. D. Miserev, D. Loss, J. Klinovaja, Instability of the ferromagnetic quantum critical point in strongly interacting 2D and 3D electron gases with arbitrary spin-orbit splitting. arXiv:2201.10995 [cond-mat.str-el] (2022).

17. J. Knorzer, M. J. A. Schuetz, G. Giedke, D. S. Wild, K. De Greve, R. Schmidt, M. D. Lukin, J. I. Cirac, Wigner crystals in two-dimensional transition-metal dichalcogenides: Spin physics and readout. *Phys. Rev. B* **101**, 125101 (2020).

18. P. Back, M. Sidler, O. Cotlet, A. Srivastava, N. Takemura, M. Kroner, A. Imamoğlu, Giant paramagnetism-induced valley polarization of electrons in charge-tunable monolayer MoSe2. *Phys. Rev. Lett.* **118**, 237404 (2017).

19. J. Li, M. Goryca, K. Yumigeta, H. Li, S. Tongay, S. A. Crooker, Valley relaxation of resident electrons and holes in a monolayer semiconductor: Dependence on carrier density and the role of substrate-induced disorder. *Phys. Rev. Mater.* **5**, 044001 (2021).

20. M. Ersfeld, F. Volmer, L. Rathmann, L. Kotewitz, M. Heithoff, M. Lohmann, B. Yang, K. Watanabe, T. Taniguchi, L. Bartels, J. Shi, C. Stampfer, B. Beschoten, Unveiling valley lifetimes of free charge carriers in monolayer WSe2. *Nano Lett.* **20**, 3147–3154 (2020).

21. C. Robert, S. Park, F. Cadiz, L. Lombez, L. Ren, H. Tornatzky, A. Rowe, D. Paget, F. Sirotti, M. Yang, D. Van Tuan, T. Taniguchi, B. Urbaszek, K. Watanabe, T. Amand, H. Dery, X. Marie, Spin/valley pumping of resident electrons in WSe_2 and WS_2 monolayers. *Nat. Commun.* **12**, 5455 (2021).

22. G. Plechinger, P. Nagler, A. Arora, R. Schmidt, A. Chernikov, A. G. del Águila, P. C. M. Christianen, R. Bratschitsch, C. Schüller, T. Korn, Trion fine structure and coupled spin–valley dynamics in monolayer tungsten disulfide. *Nat. Commun.* **7**, 12715 (2016).

23. E. Courtade, M. Semina, M. Manca, M. M. Glazov, C. Robert, F. Cadiz, G. Wang, T. Taniguchi, K. Watanabe, M. Pierre, W. Escoffier, E. L. Ivchenko, P. Renucci, X. Marie, T. Amand, B. Urbaszek, Charged excitons in monolayer WSe_2: Experiment and theory. *Phys. Rev. B* **96**, 085302 (2017).

24. S.-Y. Chen, T. Goldstein, T. Taniguchi, K. Watanabe, J. Yan. Coulomb-bound four- and five-particle intervalley states in an atomically-thin semiconductor. *Nat. Commun.* **9**, 3717 (2018).

25. Z. Ye, L. Waldecker, E. Y. Ma, D. Rhodes, A. Antony, B. Kim, X.-X. Zhang, M. Deng, Y. Jiang, Z. Lu, D. Smirnov, K. Watanabe, T. Taniguchi, J. Hone, T. F. Heinz. Efficient generation of neutral and charged biexcitons in encapsulated WSe_2 monolayers. *Nat. Commun.* **9**, 3718 (2018).

26. Z. Li, T. Wang, Z. Lu, C. Jin, Y. Chen, Y. Meng, Z. Lian, T. Taniguchi, K. Watanabe, S. Zhang, D. Smirnov, S.-F. Shi. Revealing the biexciton and trion-exciton complexes in BN encapsulated WSe_2. *Nat. Commun.* **9**, 3719 (2018).

27. M. Barbone, A. R.-P. Montblanch, D. M. Kara, C. Palacios-Berraquero, A. R. Cadore, D. De Fazio, B. Pingault, E. Mostaani, H. Li, B. Chen, K. Watanabe, T. Taniguchi, S. Tongay, G. Wang, A. C. Ferrari, M. Atatüre. Charge-tuneable biexciton complexes in monolayer WSe_2. *Nat. Commun.* **9**, 3721 (2018).

28. H. Yu, G.-B. Liu, P. Gong, X. Xu, W. Yao, Dirac cones and Dirac saddle points of bright excitons in monolayer transition metal dichalcogenides. *Nat. Commun.* **5**, 3876 (2014).

29. Z. F. Wang, J. Shan, K. F. Mak, Valley- and spin-polarized Landau levels in monolayer WSe2. *Nat. Nanotechnol.* **12**, 144–149 (2017).

30. J. Li, M. Goryca, N. P. Wilson, A. V. Stier, X. Xu, S. A. Crooker, Spontaneous valley polarization of interacting carriers in a monolayer semiconductor. *Phys. Rev. Lett.* **125**, 147602 (2020).

31. J. Li, M. Goryca, J. Choi, X. D. Xu, S. A. Crooker, Many-body exciton and intervalley correlations in heavily electron-doped WSe_2 monolayers. *Nano Lett.* **22**, 426–432 (2022).

32. C. Zhang, H. Wang, W. Chan, C. Manolatou, F. Rana, Absorption of light by excitons and trions in monolayers of metal dichalcogenide MoS_2: Experiments and theory. *Phys. Rev. B* **89**, 205436 (2014).

33. M. M. Glazov, Optical properties of charged excitons in two-dimensional semiconductors. *J. Chem. Phys.* **153**, 034703 (2020).

34. J. Wang, C. Manolatou, Y. Bai, J. Hone, F. Rana, X. Zhu, Disorder of Excitons and Trions in Monolayer MoSe2. arXiv:2111.09683 [cond-mat.mes-hall] (2021).

35. C. H. Jin, J. Kim, M. I. B. Utama, E. C. Regan, H. Kleemann, H. Cai, Y. X. Shen, M. J. Shinner, A. Sengupta, K. Watanabe, T. Taniguchi, S. Tongay, A. Zettl, F. Wang, Imaging of pure spin-valley diffusion current in WS_2-WSe_2 heterostructures. *Science* **360**, 893–896 (2018).

36. J. G. Rousset, J. Papierska, W. Pacuski, A. Golnik, M. Nawrocki, W. Stefanowicz, S. Stefanowicz, M. Sawicki, R. Jakieła, T. Dietl, A. Navarro-Quezada, B. Faina, T. Li, A. Bonanni, J. Suffczyński, Relation between exciton splittings, magnetic circular dichroism, and magnetization in wurtzite $Ga_{1-x}Fe_xN$. *Phys. Rev. B* **88**, 115208 (2013).

37. Z. Fei, B. Huang, P. Malinowski, W. Wang, T. Song, J. Sanchez, W. Yao, D. Xiao, X. Zhu, A. F. May, W. Wu, D. H. Cobden, J. H. Chu, X. Xu, Two-dimensional itinerant ferromagnetism in atomically thin Fe_3GeTe_2. *Nat. Mater.* **17**, 778–782 (2018).

38. K. Huang, *Statistical Mechanics* (Wiley, 1963).

39. C. D. Stanciu, F. Hansteen, A. V. Kimel, A. Kirilyuk, A. Tsukamoto, A. Itoh, T. Rasing, All-optical magnetic recording with circularly polarized light. *Phys. Rev. Lett.* **99**, 047601 (2007).

40. A. Kirilyuk, A. V. Kimel, T. Rasing, Ultrafast optical manipulation of magnetic order. *Rev. Mod. Phys.* **82**, 2731(2010), 2784.

41. C. H. Lambert, S. Mangin, B. S. D. C. S. Varaprasad, Y. K. Takahashi, M. Hehn, M. Cinchetti, G. Malinowski, K. Hono, Y. Fainman, M. Aeschlimann, E. E. Fullerton, All-optical control of ferromagnetic thin films and nanostructures. *Science* **345**, 1337–1340 (2014).

42. A. V. Kimel, M. Li, Writing magnetic memory with ultrashort light pulses. *Nat. Rev. Mater.* **4**, 189–200 (2019).

43. R. K. Kawakami, Spin amplification by controlled symmetry breaking for spin-based logic. *2D Mater.* **2**, 034001 (2015).

44. B. Huang, G. Clark, E. Navarro-Moratalla, D. R. Klein, R. Cheng, K. L. Seyler, D. Zhong, E. Schmidgall, M. A. McGuire, D. H. Cobden, W. Yao, D. Xiao, P. Jarillo-Herrero, X. Xu, Layer-dependent ferromagnetism in a Van der Waals crystal down to the monolayer limit. *Nature* **546**, 270–273 (2017).

45. C. Gong, L. Li, Z. Li, H. Ji, A. Stern, Y. Xia, T. Cao, W. Bao, C. Wang, Y. Wang, Z. Q. Qiu, R. J. Cava, S. G. Louie, J. Xia, X. Zhang, Discovery of intrinsic ferromagnetism in two-dimensional Van der Waals crystals. *Nature* **546**, 265–269 (2017).

46. K. F. Mak, J. Shan, D. C. Ralph, Probing and controlling magnetic states in 2D layered magnetic materials. *Nat. Rev. Phys.* **1**, 646–661 (2019).

47. F. Liu, T. Makino, T. Yamasaki, K. Ueno, A. Tsukazaki, T. Fukumura, Y. Kong, M. Kawasaki, Ultrafast time-resolved faraday rotation in EuO thin films. *Phys. Rev. Lett.* **108**, 257401(2012).

48. Q. C. Sun, T. Song, E. Anderson, A. Brunner, J. Förster, T. Shalomayeva, T. Taniguchi, K. Watanabe, J. Gräfe, R. Stöhr, X. Xu, J. Wrachtrup, Magnetic domains and domain wall pinning in atomically thin $CrBr_3$ revealed by nanoscale imaging. *Nat. Commun.* **12**, 1989 (2021).

49. P. Maletinsky, S. Hong, M. S. Grinolds, B. Hausmann, M. D. Lukin, R. L. Walsworth, M. Loncar, A. Yacoby, A robust scanning diamond sensor for nanoscale imaging with single nitrogen-vacancy centres. *Nat. Nanotechnol.* **7**, 320–324 (2012).

50. T. C. Song, Q. C. Sun, E. Anderson, C. Wang, J. M. Qian, T. Taniguchi, K. Watanabe, M. A. McGuire, R. Stohr, D. Xiao, T. Cao, J. Wrachtrup, X. D. Xu, Direct visualization of magnetic domains and moiré magnetism in twisted 2D magnets. *Science* **374**, 1140–1144 (2021).

51. X. Z. Yu, Y. Onose, N. Kanazawa, J. H. Park, J. H. Han, Y. Matsui, N. Nagaosa, Y. Tokura, Real-space observation of a two-dimensional skyrmion crystal. *Nature* **465**, 901–904 (2010).

52. J. R. Schaibley, H. Yu, G. Clark, P. Rivera, J. S. Ross, K. L. Seyler, W. Yao, X. Xu, Valleytronics in 2D materials. *Nat. Rev. Mater.* **1**, 16055 (2016).

53. K. F. Mak, J. Shan, Photonics and optoelectronics of 2D semiconductor transition metal dichalcogenides. *Nat. Photonics* **10**, 216–226 (2016).

54. Q. H. Wang, K. Kalantar-Zadeh, A. Kis, J. N. Coleman, M. S. Strano, Electronics and optoelectronics of two-dimensional transition metal dichalcogenides. *Nat. Nanotechnol.* **7**, 699–712 (2012).

55. J. F. Sierra, J. Fabian, R. K. Kawakami, S. Roche, S. O. Valenzuela, Van der Waals heterostructures for spintronics and opto-spintronics. *Nat. Nanotechnol.* **16**, 856–868 (2021).

56. A. Figotin, I. Vitebsky, Nonreciprocal magnetic photonic crystals. *Phys. Rev. E* ***63***, 066609 (2001).

57. L. Bi, J. Hu, P. Jiang, D. H. Kim, G. F. Dionne, L. C. Kimerling, C. A. Ross, On-chip optical isolation in monolithically integrated non-reciprocal optical resonators. *Nat. Photonics* **5**, 758–762 (2011).

58. R. Shreiner, K. Hao, A. Butcher, A. A. High, Electrically controllable chirality in a nanophotonic interface with a two-dimensional semiconductor. *Nat. Photonics* **16**, 330–336 (2022).

59. B. J. Shastri, A. N. Tait, T. Ferreira de Lima, W. H. P. Pernice, H. Bhaskaran, C. D. Wright, P. R. Prucnal, Photonics for artificial intelligence and neuromorphic computing. *Nat. Photonics* **15**, 102–114 (2021).

60. A. Laturia, M. L. Van de Put, W. G. Vandenberghe, Dielectric properties of hexagonal boron nitride and transition metal dichalcogenides: From monolayer to bulk. *Npj 2D Mater. Appl.* **2**, 6 (2018).

61. H. Kataura, Y. Kumazawa, Y. Maniwa, I. Umezu, S. Suzuki, Y. Ohtsuka, Y. Achiba, Optical properties of single-wall carbon nanotubes. *Synth. Met.* **103**, 2555–2558 (1999).

62. E. H. Hasdeo, A. R. T. Nugraha, M. S. Dresselhaus, R. Saito, Breit-Wigner-Fano line shapes in Raman spectra of graphene. *Phys. Rev. B* **90**, 245140 (2014).
